# Supplementary material for: Cortico-striatal action control inherent of opponent cognitive-motivational styles
Source: eLife. 2025 Feb 19;13:RP100988. doi: 10.7554/eLife.100988 (PMC11839163; doi:10.7554/eLife.100988)
Supplement: MDAR checklist [file elife-100988-mdarchecklist1.docx]

**Materials Design Analysis Reporting (MDAR)**

**Checklist for Authors**

The [MDAR framework](https://osf.io/xfpn4/) establishes a minimum set of requirements in transparent reporting mainly applicable to studies in the life sciences.

*eLife* asks authors to **provide detailed information within their article** to facilitate the interpretation and replication of their work. Authors can also upload supporting materials to comply with relevant reporting guidelines for health-related research (see [EQUATOR Network](http://www.equator-network.org/%20)), life science research (see the [BioSharing Information Resource](http://biosharing.org/)), or animal research (see the [ARRIVE Guidelines](http://www.plosbiology.org/article/info:doi/10.1371/journal.pbio.1000412) and the [STRANGE Framework](https://doi.org/10.1038/d41586-020-01751-5); for details, see *eLife*’s [Journal Policies](https://reviewer.elifesciences.org/author-guide/journal-policies)). Where applicable, authors should refer to any relevant reporting standards materials in this form.

For all that apply, please note **where in the article** the information is provided. Please note that we also collect information about data availability and ethics in the submission form.

**Materials:**

| **Newly created materials** | **Indicate where provided: section/figure legend** | **N/A** |
| --- | --- | --- |
| The manuscript includes a dedicated "materials availability statement" providing transparent disclosure about availability of newly created materials including details on how materials can be accessed and describing any restrictions on access. |  | No newly created materials generated in this study |
|  |  |  |
| **Antibodies** | **Indicate where provided: section/figure legend** | **N/A** |
| For commercial reagents, provide supplier name, catalogue number and [RRID](https://scicrunch.org/resources), if available. | Materials and methods. Visualization and quantification of GFP/mCherry-expressing neurons. Rabbit anti-mCherry, ab167453, Abcam; 1:500. Donkey anti-rabbit conjugated to Alexa 594, PIA32754, Invitrogen, 1:500. |  |
|  |  |  |
| **DNA and RNA sequences** | **Indicate where provided: section/figure legend** | **N/A** |
| Short novel DNA or RNA including primers, probes: Sequences should be included or deposited in a public repository. | Materials and methods. Cre-dependent plasmid pAAV-hSyn-DIO-hM4D(Gi)-mCherry (AddGene #44362-AAV8; titer of 2.1x1013 GC/mL). Cre-dependent control plasmid pAAV-hSyn-DIO-mCherry (AddGene #50459-AAV8; titer of 2.2x1013 GC/mL). Retrogradely transported Cre-expressing plasmid pENN-rAAV-hSyn-HI-eGFP-Cre-WPRE-SV40 (AddGene #105540-AAVrg; titer of 1.9x1013 GC/mL). | No novel primers or probes were generated for this study. All primers and probes used were commercially available. |
|  |  |  |
| **Cell materials** | **Indicate where provided: section/figure legend** | **N/A** |
| Cell lines: Provide species information, strain. Provide accession number in repository OR supplier name, catalog number, clone number, OR RRID. |  | No cell lines were used in this study. |
| Primary cultures: Provide species, strain, sex of origin, genetic modification status. |  | No primary cultures were used in this study. |
|  |  |  |
| **Experimental animals** | **Indicate where provided: section/figure legend** | **N/A** |
| Laboratory animals or Model organisms: Provide species, strain, sex, age, genetic modification status. Provide accession number in repository OR supplier name, catalog number, clone number, OR RRID. | Materials and methods. Subjects. 378 Sprague Dawley rats (215 females; 250-500 g; obtained from Inotiv, West Lafayette, IN, and Taconic, Rensselaer, NY). |  |
| Animal observed in or captured from the field: Provide species, sex, and age where possible. |  | Field animals were not observed in this study. |
|  |  |  |
| **Plants and microbes** | **Indicate where provided: section/figure legend** | **N/A** |
| Plants: provide species and strain, ecotype and cultivar where relevant, unique accession number if available, and source (including location for collected wild specimens). |  | N/A |
| Microbes: provide species and strain, unique accession number if available, and source. |  | N/A |
|  |  |  |
| **Human research participants** | **Indicate where provided: section/figure legend) or state if these demographics were not collected** | **N/A** |
| If collected and within the bounds of privacy constraints report on age, sex, gender and ethnicity for all study participants. |  | **N/A** |

**Design:**

| **Study protocol** | **Indicate where provided: section/figure legend** | **N/A** |
| --- | --- | --- |
| If the study protocol has been pre-registered, provide DOI. For clinical trials, provide the trial registration number OR cite DOI. |  | N/A |
|  |  |  |
| **Laboratory protocol** | **Indicate where provided: section/figure legend** | **N/A** |
| Provide DOI OR other citation details if detailed step-by-step protocols are available. |  | N/A |
|  |  |  |
| **Experimental study design (statistics details) *** | | |
| **For in vivo studies: State whether and how the following have been done** | **Indicate where provided: section/figure legend. If it could have been done, but was not, write “not done”** | **N/A** |
| Sample size determination | Table 1. Not done, because final sample sizes depended in part on the results of behavioral screening (Pavlovian Conditioned Approach). |  |
| Randomisation | Materials and methods. Training Regimen. The modality of the turn and stop cues (e.g., auditory vs. visual) was pseudo-randomized across rats. |  |
| Blinding |  | Undergraduate research assistants conducting behavioral tests were blinded to both the phenotype of the animals and treatment (not explicitly mentioned in the manuscript). |
| Inclusion/exclusion criteria | Materials and methods. Amperometry data processing and analysis of glutamate peaks. Inclusion and exclusion criteria are detailed in this section. Inclusion/Exclusion Criteria: Glutamate signals were included if the electrode met calibration criteria, had accurate placement, and was free from major interference. Data were processed using MATLAB’s PeakDet function, with peaks defined as those exceeding 3 SDs above baseline and separated by at least 1 SD. For adjacent peaks, the highest point was used. |  |
|  |  |  |
| **Sample definition and in-laboratory replication** | **Indicate where provided: section/figure legend** | **N/A** |
| State number of times the experiment was replicated in the laboratory. | Materials and methods. Subjects. Four separate cohorts of rats were recorded from across 19 months. |  |
| Define whether data describe technical or biological replicates. | The experiment was conducted with four independent cohorts of animals (biological replicates) with multiple recordings were conducted from each animal (technical replicates). |  |
|  |  |  |
| **Ethics** | **Indicate where provided: section/submission form** | **N/A** |
| Studies involving human participants: State details of authority granting ethics approval (IRB or equivalent committee(s), provide reference number for approval. |  | N/A |
| Studies involving experimental animals: State details of authority granting ethics approval (IRB or equivalent committee(s), provide reference number for approval. | Materials and methods. All experimental procedures were approved by the University Committee on the Use and Care of Animals at the University of Michigan (protocol # PRO00010749 and PRO00011037) and carried out in laboratories accredited by the Association for Assessment and Accreditation of Laboratory Animal Care (AAALAC; Unit # 000285; PHS assurance # D16-00072 (A3114-01). |  |
| Studies involving specimen and field samples: State if relevant permits obtained, provide details of authority approving study; if none were required, explain why. |  | N/A |
|  |  |  |
| **Dual Use Research of Concern (DURC)** | **Indicate where provided: section/submission form** | **N/A** |
| If study is subject to dual use research of concern regulations, state the authority granting approval and reference number for the regulatory approval. |  | N/A |

**Analysis:**

| **Attrition** | **Indicate where provided: section/figure legend** | **N/A** |
| --- | --- | --- |
| Describe whether exclusion criteria were pre-established. Report if sample or data points were omitted from analysis. If yes, report if this was due to attrition or intentional exclusion and provide justification. | Materials and methods. Amperometry data processing and analysis of glutamate peaks. Pre-established inclusion and exclusion criteria are detailed in this section. Additionally, the data from one rat that developed seizures after the 2nd day of recordings were completely excluded from the final analyses. |  |
|  |  |  |
| **Statistics** | **Indicate where provided: section/figure legend** | **N/A** |
| Describe statistical tests used and justify choice of tests. | Materials and methods. Experimental design and statistical analyses. Chi-square tests assessed the effects of sex and vendor on PCA score distributions. Baseline CTTT performance was analyzed with repeated measures ANOVA, and individual values, means, and 95% confidence intervals (CI) were plotted. ANOVAs and linear mixed effects models (LMMs) with restricted maximum likelihood estimation were performed using SPSS and GraphPad Prism. LMMs accounted for varying sample sizes and repeated measures, with phenotype as a between-subjects factor and subject identifier as a random intercept. Bonferroni corrections were applied for post hoc comparisons. Significant interactions were further analyzed with repeated measures ANOVA (Huynh–Feldt correction for sphericity violations). For glutamate concentration data, graphs show individual values, estimated marginal means (EMMs), and 95% CI. Chi-square tests also analyzed phenotypic frequencies of traces with one to three peaks. Contingency table analyses, including Fisher’s exact test, compared proportions of turns and misses based on peak concentrations and glutamate peaks. The effects of CNO and vehicle on cued turns and stops were analyzed using repeated measures ANOVA with Tukey’s post hoc test. Exact p-values and effect sizes (ηp2) were reported. |  |
|  |  |  |
| **Data availability** | **Indicate where provided: section/submission form** | **N/A** |
| For newly created and reused datasets, the manuscript includes a data availability statement that provides details for access (or notes restrictions on access). | *The datasets generated and analyzed for this study are publicly available in Dryad at* [*https://doi.org/10.5061/dryad.8w9ghx3z1*](https://doi.org/10.5061/dryad.8w9ghx3z1)*."* |  |
| When newly created datasets are publicly available, provide accession number in repository OR DOI and licensing details where available. |  |  |
| If reused data is publicly available provide accession number in repository OR DOI, OR URL, OR citation. |  |  |
|  |  |  |
| **Code availability** | **Indicate where provided: section/figure legend** | **N/A** |
| For any computer code/software/mathematical algorithms essential for replicating the main findings of the study, whether newly generated or re-used, the manuscript includes a data availability statement that provides details for access or notes restrictions. |  | Newly generated code was used to import amperometry CSV output into MATLAB, process background current, convert to glutamate concentrations using calibration slopes, and baseline-correct the data. Peak detection was performed using the publicly available PeakDet MATLAB function. Full details of the code used are available upon request. |
| Where newly generated code is publicly available, provide accession number in repository, OR DOI OR URL and licensing details where available. State any restrictions on code availability or accessibility. |  | Code is not publicly  available but can be provided upon request. |
| If reused code is publicly available provide accession number in repository OR DOI OR URL, OR citation. | Peakdet was used in the study and can be found through this URL: https://www.mathworks.com/matlabcentral/fileexchange/47264-peakdet |  |

**Reporting:**

The MDAR framework recommends adoption of discipline-specific guidelines, established and endorsed through community initiatives.

| **Adherence to community standards** | **Indicate where provided: section/figure legend** | **N/A** |
| --- | --- | --- |
| State if relevant guidelines (e.g., ICMJE, MIBBI, ARRIVE, STRANGE) have been followed, and whether a checklist (e.g., CONSORT, PRISMA, ARRIVE) is provided with the manuscript. |  | N/A |

* We provide the following guidance regarding transparent reporting and statistics; we also refer authors to [Ten common statistical mistakes to watch out for when writing or reviewing a manuscript](https://doi.org/10.7554/eLife.48175).

**Sample-size estimation**

- You should state whether an appropriate sample size was computed when the study was being designed
- You should state the statistical method of sample size computation and any required assumptions
- If no explicit power analysis was used, you should describe how you decided what sample (replicate) size (number) to use

**Replicates**

- You should report how often each experiment was performed
- You should include a definition of biological versus technical replication
- The data obtained should be provided and sufficient information should be provided to indicate the number of independent biological and/or technical replicates
- If you encountered any outliers, you should describe how these were handled
- Criteria for exclusion/inclusion of data should be clearly stated
- High-throughput sequence data should be uploaded before submission, with a private link for reviewers provided (these are available from both GEO and ArrayExpress)

**Statistical reporting**

- Statistical analysis methods should be described and justified
- Raw data should be presented in figures whenever informative to do so (typically when N per group is less than 10)
- For each experiment, you should identify the statistical tests used, exact values of N, definitions of center, methods of multiple test correction, and dispersion and precision measures (e.g., mean, median, SD, SEM, confidence intervals; and, for the major substantive results, a measure of effect size (e.g., Pearson's r, Cohen's d)
- Report exact p-values wherever possible alongside the summary statistics and 95% confidence intervals. These should be reported for all key questions and not only when the p-value is less than 0.05.

**Group allocation**

- Indicate how samples were allocated into experimental groups (in the case of clinical studies, please specify allocation to treatment method); if randomization was used, please also state if restricted randomization was applied
- Indicate if masking was used during group allocation, data collection and/or data analysis
